# Genotype-phenotype associations in neurofibromatosis type 1 (NF1): an increased risk of tumor complications in patients with *NF1* splice-site mutations?

**DOI:** 10.1186/1479-7364-6-12

**Published:** 2012-08-13

**Authors:** Adila Alkindy, Nadia Chuzhanova, Usha Kini, David N Cooper, Meena Upadhyaya

**Affiliations:** 1 Clinical Genetics Department, Sultan Qaboos University Hospital, P.O. Box 39, Al-Khod, Muscat 123, Sultanate of Oman; 2 School of Science and Technology, Nottingham Trent University, Clifton Lane, Nottingham, NG11 8NS, UK; 3 Department of Clinical Genetics, Churchill Hospital, Old Road Headington, Oxford, OX3 7LJ, UK; 4 Institute of Medical Genetics, Cardiff University, Heath Park, Cardiff, CF14 4XN, UK

**Keywords:** Neurofibromatosis type 1, Genotype-phenotype correlation, Malignant peripheral nerve sheath tumors, Brain glioma, Increased cancer susceptibility, Splice-site mutations, *NF1* gene, Gender effect

## Abstract

Neurofibromatosis type 1 (NF1) is a complex neurocutaneous disorder with an increased susceptibility to develop both benign and malignant tumors but with a wide spectrum of inter and intrafamilial clinical variability. The establishment of genotype-phenotype associations in NF1 is potentially useful for targeted therapeutic intervention but has generally been unsuccessful, apart from small subsets of molecularly defined patients. The objective of this study was to evaluate the clinical phenotype associated with the specific types of *NF1* mutation in a retrospectively recorded clinical dataset comprising 149 *NF1* mutation-known individuals from unrelated families. Each patient was assessed for ten NF1-related clinical features, including the number of café-au-lait spots, cutaneous and subcutaneous neurofibromas and the presence/absence of intertriginous skin freckling, Lisch nodules, plexiform and spinal neurofibromas, optic gliomas, other neoplasms (in particular CNS gliomas, malignant peripheral nerve sheath tumors (MPNSTs), juvenile myelomonocytic leukemia, rhabdomyosarcoma, phaechromocytoma, gastrointestinal stromal tumors, juvenile xanthogranuloma, and lipoma) and evidence of learning difficulties. Gender and age at examination were also recorded. Patients were subcategorized according to their associated *NF1* germ line mutations: frame shift deletions (52), splice-site mutations (23), nonsense mutations (36), missense mutations (32) and other types of mutation (6). A significant association was apparent between possession of a splice-site mutation and the presence of brain gliomas and MPNSTs (*p* = 0.006). If confirmed, these findings are likely to be clinically important since up to a third of NF1 patients harbor splice-site mutations. A significant influence of gender was also observed on the number of subcutaneous neurofibromas (females, *p* = 0.009) and preschool learning difficulties (females, *p* = 0.022).

## Introduction

Neurofibromatosis type 1 (NF1) (MIM #162200) [1] is a common familial cancer syndrome with a prevalence of 1 in 2,500 [2] and an autosomal dominant inheritance pattern; it is fully penetrant by 5 years of age. NF1 is a complex disorder that affects many cell types and involves multiple body systems [3]. The clinical phenotypic expression in NF1 is characterized by marked intra and interfamilial variability with many NF1 individuals only relatively mildly affected and their disease is limited to cutaneous involvement. Most NF1 patients also have mild learning difficulties. About a quarter of NF1 cases eventually develop one or more serious clinical multisystemic complications that lead to the significant morbidity and increased mortality characteristic of this disorder [4]. The many NF1-related clinical complications include neurological, cardiovascular, gastrointestinal, endocrine and orthopedic features. The overall risk of malignancy in NF1 is 5% to 15% higher than in the general population, the age of onset being earlier and with a poorer prognosis [5]. This increased incidence of malignancy primarily involves tumors of the CNS and connective tissue [5-7].

Neurofibromin, the *NF1* gene product, is a large (2,818 amino acids) [8] ubiquitously expressed protein present at low levels in most tissues, with the highest concentration found in the CNS [3,8]. The most inactivating *NF1* germ line mutations result in neurofibromin haploinsufficiency which seriously impairs its key role as a GTPase-activating protein that down-regulates most Ras protein activity in the cell [9-11]. Since Ras has a pivotal role in up-regulating cell proliferation, differentiation, survival and cell death, any functional loss of neurofibromin will result in the inappropriate and sustained activation of the Ras/Raf/ERK pathway. It is this cellular dysregulation that explains much of the NF1 clinical phenotype, especially the increased risk of benign and malignant neoplasms evident in NF1.

NF1 is however noted for the considerable inter and intrafamilial variation observed in the clinical phenotype even in patients who share the same germ line mutation. As a result, any decisions on surveillance tailored to some of these potentially treatable complications can be difficult to implement in the clinic. Successful genetic counseling is also hampered by clinical complications that are unpredictable both in severity and progression even within the same family [4]. This is particularly important in prenatal genetic counseling where the actual disease risk to the unborn infant is difficult to predict and where prenatal diagnosis creates difficulties in reproductive decision making for the families involved [4,12]. Hence, given such disease prediction and management problems, comprehensive genotype-phenotype studies are warranted.

Unfortunately, attempts to correlate the many and varied germ line *NF1* gene mutations with specific clinical features of NF1 have been largely unsuccessful [13], mainly due to the marked inter and intrafamilial variability in disease expression and the extent of the allelic heterogeneity underlying the disease. Two potential genotype-phenotype correlations that involve small subgroups of NF1 individuals have nevertheless been reported [3,14]. At least 1,347 different *NF1* gene mutations have been identified [15], many being unique to a specific individual or family [4,16]; fewer than 20% of *NF1* gene lesions are recurrent. As might be expected from the high mutation rate observed for the human *NF1* gene, almost half of all identified mutations occur *de novo*[16]. Given the marked clinical phenotypic variation associated with NF1 even in individuals carrying the same *NF1* mutation, it has been suggested that feature-specific modifier genes which are unlinked to the *NF1* locus itself, epigenetic alterations or other environmental factors may also contribute to such variable expression in NF1 [17,18].

The present retrospective study has attempted to assess whether any associated genotype-phenotype correlations could be identified in a large cohort (*N* = 149) of clinically well-characterized (but unrelated) NF1 patients with known pathogenic *NF1* mutations.

## Methods

### Patients

The present study is a retrospective analysis of the clinical data obtained from a cohort of NF1 patients collected for a previous Welsh study that characterized the *NF1* germ line mutations in patients referred from UK-wide medical genetic centers [19]. This new study was approved by the local ethical committee, with participants giving their informed consent. The study cohort comprised 149 unrelated *NF1* germ line mutation-positive patients whose associated clinical data were obtained from medical records over the past two years. The phenotypic information from each patient was generally recorded in a standardized way. This study actively excluded all patients known to harbor either a large genomic deletion [14] or the *NF1* 3-bp in-frame deletion in exon 17 [3], because both these mutation types have previously been found to have a tendency to be associated with a specific NF1 clinical phenotype.

### Patient demographics

The patient cohort was characterized by an equal gender distribution, an ethnic origin that was predominantly Caucasian, and an age distribution which ranged from 1 day old to 71 years of age (mean age, 24 years). Most patients (137, approximately 92%) were clinically diagnosed as NF1 according to the National Institutes of Health (NIH) criteria [20]; three of these patients had concurrent diagnosis of other growth and developmental syndromes; five patients (approximately 3%) were diagnosed as NF1/Noonan syndrome (MIM #601321), four (approximately 3%) with NF1, familial spinal (MIM #162210) syndrome and three (2%) with Watson syndrome (MIM #193520). Seven patients (approximately 5%) did not meet the strict NIH clinical diagnostic criteria for NF1 when first tested for *NF1* mutations, whereas 49 patients (approximately 33%) had a positive family history of NF1, 90 patients (60%) were sporadic and 10 (approximately 7%) had an unknown family history. All patient data collected were anonymized prior to analysis.

### Clinical features and associated mutations

**Figure 1:**
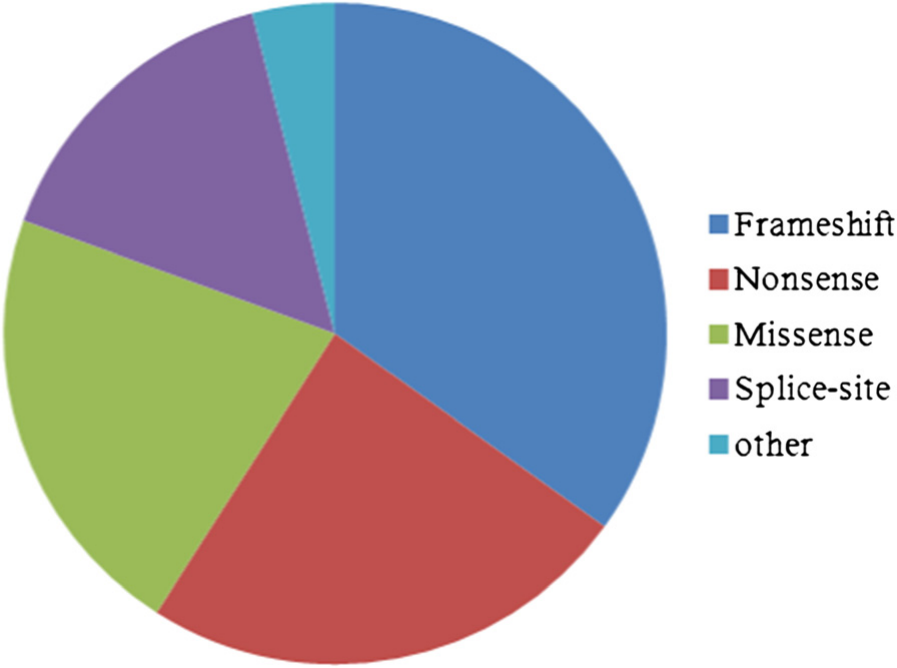
**Relative proportion of the different types of mutation among the germ line *NF1 *mutations identified.**

Ten major NF1 clinical features were recorded for each patient, alongside patient age and gender, and the associated *NF1* germ line mutation. For the purposes of this analysis, patients were divided into three age groups: <6, 6 to 19 and >19 years. The germ line *NF1* mutations were recorded in terms of the type and specificity of the mutation, its genic location, and the predicted protein change. These mutations were further subdivided into five categories (see Figure 1): frame shift mutations (52 patients), splice-site mutations (23 patients), nonsense mutations (36 patients), missense mutations (32 patients) and six other less frequent types of mutation, including in-frame deletions (2), insertion/deletion (indel) mutations (1), single exon deletions (2) and intragenic multi-exonic deletions (1). The recorded clinical details included two numerical features: (1) the number of café-au-lait (CAL) spots, with four subgroups identified *viz*., those with no CALs (ten patients), those with CALs of sizes 1 to 5 mm (11 cases), patients with CALs of sizes 6 to 99 mm (117) and four cases with large CALs (>111 mm); and (2) the number of cutaneous neurofibromas (CNFs) and subcutaneous neurofibromas (SCNFs), with patients subgrouped into those with no neurofibromas, those with only 2 to 5 CNFs, cases with 6 to 99 CNFs and SCNFs, those with 100 to 500 CNFs and SCNFs and those with >500 CNFs. Seven additional binary (presence or absence) clinical features were also examined, including skin freckling, Lisch nodules of the iris, plexiform neurofibromas (PNF), spinal neurofibromas (SpNF), optic gliomas, rare tumors known to be commonly associated with NF1 (including CNS gliomas, malignant peripheral nerve sheath tumors (MPNSTs), juvenile myelomonocytic leukemia (JMML), rhabdomyosarcoma, phaechromocytoma, gastrointestinal stromal tumors (GIST), juvenile xanthogranuloma and lipoma) and any other type of tumor recorded, and evidence of preschool learning difficulties.

## Results

A multinomial logistic regression analysis was performed using mutation type, age group and gender as covariates, and the absence of clinical features as a reference (control) category. The results of this analysis, following removal of outliers, revealed a highly significant effect of the mutation type (specifically splice-site mutations) on the number of CALs, both with 1 to 5 CALs (*p* = 1.8 × 10^−27^) and 6 to 99 CALs (*p* = 8.5 × 10^−43^), as well as for skin freckling (*p* = 0.028) and neoplasms (*p* = 0.006). CNS gliomas and MPNST contributed to almost half of this group of neoplasms (see Table 1).

**Table 1:** **Prevalence of neoplasms in a cohort (*N*** **= 149 with known NF1 mutation) of clinically well-characterized NF1 patients**

**Type of neoplasm**	**Number of neoplasms**^**a**^** reported (%)**	
CNS gliomas (excluding optic gliomas)	12	8
Malignant peripheral nerve sheath tumor	8	5.5
Rhabdomyosarcoma (prostate)	1	0.7
Medullary thyroid carcinoma	2	1.3
Gastrointestinal stromal tumors	2	1.3
Multiple internal neurofibromas	3	2
Phaechromocytoma	3(1B/L)	2
Colorectal carcinoma at 71 years	1	0.7
Breast cancer - ductal type at 34 and 40 years	2	1.3
Glomus tumor	2	1.3
Xanthogranuloma	2	1.3
Lipoma	4	2.7
Juvenile myelomonocytic leukemia	0	0

^a^Some of the patients were found to have more than one type of tumor.

For the 6 to 19 years age group, a significant association was noted between the presence of a splice-site mutation and both CNF and SCNF numbers (6 to 99) (*p* = 0.002 and *p* = 0.004 respectively) and the presence of preschool learning difficulties (*p* = 0.005). A significant gender effect (female) was also observed with respect to SCNF number (6 to 99) (female, *p* = 0.009) and preschool learning difficulties (*p* = 0.022). This study therefore indicates a potential novel NF1 genotype-phenotype correlation, with *NF1* splice-site mutations being significantly associated (*p* = 0.006) with the tendency to develop tumors mostly composed of MPNSTs and CNS gliomas.

### Unusual cases

Several patients did not fulfill the strict NIH diagnostic criteria for NF1 even although they had been found to harbor *NF1* gene mutations. The NIH diagnostic criteria remain the ‘gold standard’ for NF1 diagnosis, the isolated occurrence of many of the NF1 features and complications being thought to be simply sporadic events [4,21]. Thus, *NF1* mutation testing is not routinely carried out unless there is a high index of suspicion of the disorder. Our findings indicate that such *NF1* gene testing, which usually serves to confirm the clinical diagnosis in >95% of cases [4,16], should also be considered in the context of atypical cases. Some examples from the present patient cohort with identified *NF1* mutations include the following:

(1) An 18-year-old female presenting with an MPNST of the breast but with no other characteristic NF1 features or complications. Several of her family members were also reported to have had breast cancer but they lacked other features of NF1.

(2) A 10-year-old boy with a unilateral optic glioma, a few small CALs and mild learning difficulties but with a negative family history of NF1.

(3) NF1 patients with hypertrophic cardiomyopathy (HCM). In the study cohort, two cases of a father and his 29-year-old son and a 71-year-old male were noted to have symptomatic HCM which was diagnosed by echocardiogram and manifested as cardiovascular-associated NF1 complications. Echocardiographic findings of a septal to posterior left ventricular free wall ratio greater than 1.5 (suggestive of hypertrophic cardiomyopathy) were noted in at least 4% of NF1 patients [22,23] in one study. However, none of the 2,322 NF1 individuals retrospectively assessed for cardiovascular malformation in the National Neurofibromatosis Foundation International Database had HCM [24], suggesting that routine echocardiography should form part of the surveillance regimen in NF1. At least four other single reports of the cooccurrence of HCM and NF1 [25-28] have appeared in the literature. Cardiac hypertrophy is the most common risk factor contributing to cardiovascular mortality and morbidity in NF1 patients [29]. Congenital heart disease and cardiomyopathy are also a common accompaniment of several growth and development syndromes affecting the RASopathies [30,31]. Cardiomyopathy in these syndromes arises from increased signaling through Erk1/2 and mTOR complex 1 [29]. Neurofibromin is known to act in modulating the epithelial-mesenchymal transformation and proliferation in the developing heart by down-regulating RAS activity. In the absence of functional neurofibromin, mouse embryonic hearts develop an over-abundance of endocardial cushion which may obstruct blood outflow in some cases as a result of cellular hyperproliferation and a lack of apoptosis [32-34]. The cardiac-specific *Nf1* knockout mice, combined with genetic rescue, suggest that neurofibromin is an important regulator of Ras signaling in cardiac myocytes, and that Ras activation can lead to progressive cardiac hypertrophy with associated pathological changes in adult mice [32,35,36].

(4) NF1 patients with absent CNF and SCNF. In the study cohort, 18 (12%) unrelated adult patients lacked peripheral and spinal neurofibromas, and only three patients had identifiable plexiform neurofibromas (see Table 2). All patients fulfilled the strict NIH diagnostic criteria for NF1 except for three patients who manifested only CALs and mild learning difficulties. In general, all these patients exhibited pigmentary changes of NF1 with very few NF1-related complications and had a milder clinical phenotype [3,4]. The types of mutations reported in this group were missense (six patients), nonsense (four) and microdeletions/microinsertions (seven). Only a single patient had a splice-site mutation and severe learning difficulties. Our finding is consistent with the previous observations, suggesting that the clinical phenotype of deceased NF1 patients is characterized by fewer CALs (albeit nonsignificant) and significantly more neurofibromas [6].

**Table 2:** **Summary of clinical features and germ line *NF1 *mutation type in 18 patients lacking peripheral neurofibromas**

**Patient ID**	**Sex/age (years)**	**Mutation type**	**NIH**	**CNF/ SCNF**	**PNF**	**SpNF**	**6-99 CALs**	**Skin freckling**	**Other NF1 features/ complications**
S149	F/24	Missense K1423E	Y	0	0	0	Y	B/L axilla	Low grade MPNST
S174	M/adult	Missense W1931R	Y	0	0	0	Y	B/L axilla	none
462	F/43	Missense F1193C	Y	0	0	0	Y	B/L axilla	Offspring-same phenotype
482	F/39	Missense W837R	Y	0	Y	0	Y	B/L axilla	GIST tumor
2198	M/22	Missense M11491	N	0	0	0	Y	N	Mild learning difficulties/ CALs only^a^
2444	M/24	Missense L1812P	Y	0	0	0	Y	B/L axilla/groin	CNS glioma
2483	F/29	Nonsense R1748X	Y	0	0	0	Y	extensive	none
2512	M/22	Nonsense R416X	Y	O	Y	0	Y	Submammary	none
2070	F/19	Nonsense R461X	Y	0	0	0	Y	B/L axilla	Unilateral optic glioma
2133	M/51	Nonsense K1517X	N	0	0	0	Y	N	CALs only^a^
2199	F/27	Frameshift c.6403insGA	N	0	0	0	Y	N	Mild learning difficulties/ CALs only^a^
2277	F/34	Frameshift c.3721-3722 ins A	Y	0	Y	0	Y	N	none
2278	M/39	Frameshift c.7892-7893delAA	Y	0	0	0	Y	B/L axilla	Mild learning difficulties
1663	M/25	Frameshift c.495delTGTT	Y	0	0	0	Y	B/L axilla	none
26445	F/18	Frameshift c.5406insT	Y	0	0	0	Y	Extensive	Mild learning difficulties
870	F/18	Frameshift c.6219 delT	Y	0	0	0	Y	B/L axilla/trunk	Bilateral optic glioma
2276	F/20	Frameshift c.6791insA	Y	0	0	0	Y	B/L axilla	none
2023	F/22	Splice-site c.589-2 A > G	?	0	0	0	Y	?	Severe learning difficulties

^a^Some of the patients were found to have more than one type of tumor.

## Discussion

NF1 patients with germ line *NF1* splice-site mutations were found to have an increased tendency to develop MPNSTs and CNS gliomas by comparison with patients harboring other mutation types, e.g. microinsertions, microdeletions, nonsense and missense mutations. Up to 30% of all NF1 patients carry germ line *NF1* splice-site mutations, and such mutations often lead to the synthesis of a truncated form of neurofibromin [4,16]. In the present NF1 patient cohort, however, only 23 individuals (15%) carried splice-site mutations. This comparatively low frequency could be explained by the fact that the current analysis was performed on genomic DNA (rather than cDNA), and some of the underlying splicing mutations (resulting from deep intronic mutations) could have been missed if they did not occur in the immediate vicinity of a splice site. Moreover, some of the missense or nonsense mutations identified could have exerted an effect on splicing. Despite the small patient number, our analyses identified a significant correlation between the presence of a splice-site mutation and the occurrence of neoplasms (*p* = 0.006), mostly brain and connective tissue tumors. This initial finding clearly merits further clinical investigation in a much larger mutation-known NF1 patient population. A multicenter study and prospective data collection, based on comprehensive clinical examination, would be necessary to substantiate this finding. If our initial conclusion is supported by further analysis, it could imply that the wider application of RNA-based analyses in NF1 patient screening (in order to detect splice-site mutations with high efficiency) [33] might be helpful in predicting which patients would benefit the most from regular clinical surveillance.

Of all the characteristic features of NF1, the presence of benign and malignant neoplasm contributes the most to the morbidity and mortality experienced by many NF1 individuals; indeed, the overall cancer risk associated with NF1 has been reported to be higher than in the general population in several NF1 studies [5-7], owing to markedly increased risks of brain and connective tissue tumors. Indeed, malignancy, alongside other tumor-related neurological complications, is the most frequent cause of death in NF1 patients, resulting in a reduced life expectancy of some 8 to 15 years as compared to the general population [5,7,27]. The predominance of cancer among causes of death in NF1 is consistent in most studies [5-7,34,37] and contributes disproportionately to the mortality in younger age groups (less than 40 years). This increased risk of malignancy in NF1 individuals is a cause of major concern for families and is difficult to address during genetic counseling.

If our finding of an association between the possession of an *NF1* splice-site mutation and the likelihood of tumor occurrence is borne out by future work, this group of patients should undergo rigorous clinical surveillance in order to facilitate early diagnosis and permit early management of any tumor-related complications as they arise. Although the types of tumors observed in our patient cohort were heterogeneous, they were similar to the spectrum of tumors reported in many NF1 patients, being mainly brain gliomas and MPNSTs [5-7,38] which comprised almost half of the neoplasms in our cohort. The prevalence of types of tumors other than the brain gliomas and MPNSTs reported in this cohort was very low, as noted in other studies [7]. A moderately increased susceptibility to develop breast cancer in female NF1 patients before the age of 50 years has previously been documented [39]. However, only 2/74 female (2.7%) cases in our cohort developed ductal type breast cancer between the ages of 34 and 41 years. Phaechromocytomas and catecholamine-secreting tumors of the neural crest are rare tumors in the general population and occur in 0.1% to 5.7% [40] of patients with NF1. In a recent study, a higher prevalence of 14.7% was noted [41]. At least three patients (2%) in our cohort had phaechromocytoma, whereas two patients had medullary thyroid cancer, one of these in association with bilateral phaechromocytoma as part of multiple endocrine neoplasia type 2A (MEN2A). The coexistence of MEN2A and NF1 (the neurocristopathies) has rarely been reported, suggesting that NF1 patients with phaechromocytoma should be thoroughly examined for clinical evidence of other phaechromocytoma-related syndromes. All three cases reported in the literature (including our own case) had identifiable germ line mutations in the *NF1* gene, but not in the *RET* proto-oncogene which is a known cause of MEN2A [42]. Colorectal carcinomas have been previously noted as a cause of death in NF1 [7]. Only one case of adenocarcinoma of the colon at the age of 71 was noted in our cohort and this could be a sporadic occurrence. GIST are mesenchymal tumors of the gastrointestinal tract and are commonly associated with NF1 and usually with a good prognosis [43,44]. We report two such tumors in our cohort. Glomus tumors are small, benign but painful tumors and are usually associated with NF1 and considered as part of the tumor spectrum of NF1 [45]. Two cases of multiple glomus tumors were reported in our cohort. Rhabdomyosarcomas are pediatric neoplasms which are more common in children with NF1 than in the general population [46]. One case of a child with rhabdomyosarcoma of the prostate was noted in the study cohort. Multiple subcutaneous lipomas [47] have been noted in one patient with NF1. However, solitary lipomas are also frequent in the general population, and the presence of these isolated lipomas in four of our patients could be purely coincidental. The presence of xanthogranulomas in pediatric NF1 cases may be related to the possible development of JMML [48], but none of the patients in our cohort had JMML. The prevalence of xanthogranulomas in the general population of normal children is quite high (1% to 2%), but these are rarely associated with systemic manifestations [48].

Our observation of a gender effect in NF1 has not been previously reported; despite the very similar sex distribution in our patient cohort, a disproportionate number of females exhibited learning difficulties (*p* = 0.022). Female NF1 patients were also found to be significantly more likely to manifest SCNFs (*p* = 0.009). This observation may relate to the differential effect that female steroid hormones appear to have on the development of CNFs and SCNFs, as previously noted in the peri-pubertal and pregnant subpopulations of affected NF1 individuals [49,50]. Peripheral neurofibromas have also been reported to express progesterone receptors [49,50] and this could explain the observed gender effect. SCNFs rarely transform into malignant tumors, but their presence could be an expression of a more aggressive disease [5,51]. A disproportionate number of SCNFs in a cohort of NF1 female patients could indicate a worse prognosis in females in comparison to males, as indicated in some studies [7,34,51].

## Conclusions

Our study suggests a novel potential NF1 genotype-phenotype correlation, with *NF1* splice-site mutations being associated with an increased tendency to develop neoplasms, mostly composed of CNS gliomas and MPNSTs. This finding appears to constitute a novel genotype-phenotype correlation that has the potential to be clinically useful. Since up to one third of all NF1 patients harbor splice-site mutations, the early detection of this type of lesion might be helpful in predicting which patients would benefit the most from regular clinical surveillance, thereby improving their survival. Our study also supports previous observations of a gender effect on mortality and morbidity associated with NF1; significantly, more female patients exhibited learning difficulties and were found to be more likely to possess subcutaneous neurofibromas than their male counterparts.

## Competing interests

The authors declare that they have no competing interests.

## Authors’ contributions

AA collected the clinical data and wrote the first draft of this paper. NC performed the bioinformatic data analysis, UK provided cinical data, DNC helped with the writing of the manuscript and MU provided the mutational data, helped with the writing of manuscript. All authors read and approved the final manuscript.
